# Effects of long working hours and shift work during pregnancy on obstetric and perinatal outcomes: A large prospective cohort study—Japan Environment and Children’s Study

**DOI:** 10.1111/birt.12463

**Published:** 2019-10-31

**Authors:** Nobuhiro Suzumori, Takeshi Ebara, Taro Matsuki, Yasuyuki Yamada, Sayaka Kato, Toyonori Omori, Shinji Saitoh, Michihiro Kamijima, Mayumi Sugiura‐Ogasawara, the Japan Environment & Children’s Study Group

**Affiliations:** ^1^ Department of Obstetrics and Gynecology Graduate School of Medical Sciences Nagoya City University Nagoya Japan; ^2^ Department of Occupational and Environmental Health Graduate School of Medical Sciences Nagoya City University Nagoya Japan; ^3^ Graduate School of Health and Sports Science Juntendo University Chiba Japan; ^4^ Department of Pediatrics and Neonatology Graduate School of Medical Sciences Nagoya City University Nagoya Japan; ^5^ Department of Health Care Policy and Management Graduate School of Medical Sciences Nagoya City University Nagoya Japan; ^6^ National Center for Child Health and Development Tokyo Japan

**Keywords:** complications, fetal growth, night shifts, pregnancy, working time

## Abstract

**Background:**

The work patterns of pregnant women may be related to adverse obstetric and perinatal outcomes. This study aimed to clarify the effects of weekly working time according to frequencies of night shifts during pregnancy on adverse outcomes in Japan.

**Methods:**

The Japan Environment and Children's Study, a prospective cohort study, was conducted in 15 regions nationwide in Japan. The study population included pregnant women with singleton pregnancies (n = 99 744). The mothers’ working hours and frequencies of night shifts during the first and the second/third trimesters were assessed using a self‐administered questionnaire. Outcome data were collected from medical transcripts.

**Results:**

Compared with nonworking women, women who worked during pregnancy had significantly increased adjusted odds ratios (aORs) of threatened miscarriage (maximum aOR: 1.47, 95% confidence interval [95% CI]: 1.26‐1.73) and of threatened preterm labor (maximum aOR: 1.63, 95% CI: 1.41‐1.87). Increased aORs were observed for hypertensive disorders of pregnancy (maximum aOR: 2.02, 95% CI: 1.39‐2.93) in women working ≥36 hours per week with night shifts, for vacuum/forceps delivery (maximum aOR: 1.34, 95% CI: 1.22‐1.48) at ≥36 hours with or without night shifts, and for small‐for‐gestational‐age babies (aOR: 1.32, 95% CI: 1.10‐1.59) at ≥46 hours with night shifts. In contrast, lower aORs were observed for gestational diabetes and meconium‐stained amniotic fluid in women working without night shifts.

**Conclusions:**

Work during pregnancy slightly increased the risks of threatened miscarriage and threatened preterm labor. Long working hours increased the risks of hypertensive disorders of pregnancy, vacuum/forceps delivery, and small‐for‐gestational‐age babies.

## 1 INTRODUCTION

Working during pregnancy may play an important role in adverse obstetric, perinatal, and children's outcomes.^1^, ^2^ Most pregnant workers are exposed to some physical activity at work. Several lines of evidence suggest that work itself does not increase the risks of pregnancy complications, although long working hours, prolonged standing, heavy lifting, or unusual workloads may pose a threat to pregnant workers.^3^ To what extent such evidence is consistently found in countries such as Japan, where the average working hours are 20% longer than those in five European countries (Germany, Denmark, Norway, the Netherlands, and France) and similar to those in the United States,^4^ is increasingly important because the number of pregnant workers has been increasing. However, large‐scale studies on the effect of working time during pregnancy on obstetric and children's outcomes are scarce in such countries.

The results of a recent retrospective cohort study suggested that fatigue from long continuous working periods plays a role in increasing the risk of adverse delivery outcomes.^5^ Stress‐dependent dysregulation of the hypothalamic‐pituitary axis affects birthweight and subsequent child growth and development.^6^, ^7^, ^8^ High levels of job strain during early pregnancy were associated with reduced birthweight, particularly in mothers working 32 or more hours per week.^2^ Furthermore, evidence has been accumulating from systematic reviews, meta‐analyses, and register‐based prospective cohort studies about obstetric outcomes of pregnant workers.^9^, ^10^, ^11^, ^12^, ^13^

With respect to shift work in women, shift workers may have increased rates of menstrual disruption and night shifts may be associated with increased rates of spontaneous abortion.^9^, ^10^, ^12^, ^13^ However, whether these results are applicable to other settings remains controversial as another study showed no significant increase in miscarriage rates among shift work nurses.^14^ Because research on the relationship between shift work and pregnancy outcomes is limited, the present study was conducted to provide a scientific basis for better work‐life balance in pregnant women and key precautions for prenatal care.

We have conducted a nationwide prospective birth cohort study, the Japan Environment and Children's Study (JECS), which was planned by the Ministry of the Environment, Government of Japan.^15^, ^16^, ^17^, ^18^, ^19^ The study comprises 104 102 registered prenatal records and the delivered children will be followed up until they reach 13 years of age to investigate the effect of environment, especially during the fetal period, on the health of babies and their subsequent growth and health. The present study assessed the association of weekly working hours and frequency of night shifts during pregnancy with pregnancy and perinatal outcomes. The purpose of this study was to clarify the effects of working time according to frequencies of night shifts on complications of pregnancy, modes of delivery, and incidence of small‐for‐gestational‐age babies in Japan.

## 2 METHODS

### 2.1 Study population

Between January 2011 and March 2014, pregnant women were recruited for the JECS. Women who (a) lived in any of the study areas selected by the 15 Regional Centers located countrywide at the time of recruitment; (b) had an expected delivery date after August 1, 2011; and (c) were capable of understanding the Japanese language and completing the self‐administered questionnaire were included in the study.^15^

**Figure 1:**
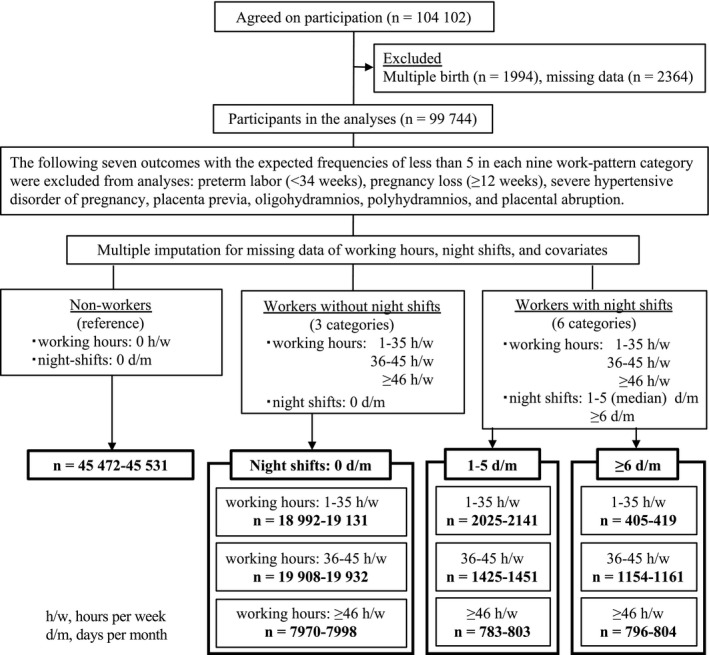
Flowchart showing the categorization of study participants by number of working hours and number of night shifts during the second/third trimesters of pregnancy, Japan, 2011‐2014. The selection process was similar for the first‐trimester analysis

This study was based on the jecs‐ag‐20160424 data set containing 104 102 records of pregnant women that was released restrictively to the researchers conducting the JECS in July 2016 (Figure 1). Overall, 1994 women with multiple pregnancies were excluded because their obstetric and perinatal outcomes were possibly affected by such pregnancies. Women with missing information on singleton or multiple pregnancies were also excluded from the analyses (n = 2364).

### 2.2 Data collection

The study participants completed questionnaires twice during their pregnancy, that is, in the first (n* = *91 029) and second/third trimesters (n* = *92 550) with the exception where some participants completed both questionnaires during the final trimester. Their medical records at the time of registration and just after delivery were transcribed by doctors, research coordinators, nurses, or midwives. The first questionnaire asked about their occupation on the awareness of their pregnancy, working hours and frequencies of night shifts at the time of answering the questionnaire, medical histories, and details of all previous pregnancies. The second questionnaire asked about working hours and the frequency of night shifts during the second/third trimester and socioeconomic characteristics. Smoking, alcohol use, and psychological distress were measured by the two questionnaires.

The medical record transcript information collected at the time of registration included maternal age, gestational weeks at registration, and maternal body weight and height at conception. The second medical record transcript included gestational weeks at miscarriage/delivery, pregnancy complications, delivery outcomes (miscarriage/induced abortion, live birth/stillbirth, single/multiple births, birthweight, normal/vacuum/forceps/cesarean birth), and perinatal outcomes.

### 2.3 Obstetric and perinatal outcomes

The obstetric outcomes evaluated in the present study were threatened miscarriage (<21 weeks of gestation), threatened preterm labor (22‐36 weeks), preterm labor (<37 and <34 weeks), mild hypertensive disorders of pregnancy (systolic blood pressure 140 to <160 mm Hg and/or diastolic blood pressure 90 to <110 mm Hg with or without proteinuria), gestational diabetes mellitus, premature rupture of the membrane, nonreassuring fetal status, oligohydramnios (amniotic fluid index <5), meconium‐stained amniotic fluid, prolonged labor over 30 hours for primipara or 15 hours for multipara during delivery, and modes of delivery. Outcomes with the expected frequencies of <5 in each of the nine work‐pattern categories were excluded from the analyses (Figure 1). Diagnoses of hypertensive disorders of pregnancy and gestational diabetes were made according to the criteria of the Japan Society of Obstetrics and Gynecology (JOGR) and the Japan Diabetes Society (JDS),^20^ respectively. The perinatal outcomes were small‐for‐gestational‐age (<10th percentile according to the Standardization Committee of Fetal Measurement of the Japanese Society of Ultrasound in Medicine) and low birthweight (<2500 g).

### 2.4 Data analysis

The participants were categorized according to the frequencies of night shifts (days per month) (first trimester: 0, 1‐6, and ≥7; second/third trimesters: 0, 1‐5, and ≥6), and working hours (hours per week) (first/second/third trimesters: 1‐35, 36‐45, and ≥46). Each grouping for night shift frequency was based on the median frequency for shift workers (first trimester: 6 days per month; second/third trimesters: 5 days per month), whereas that for working hours was based on the limit (40 hours per week) prescribed by the Labor Standard Act in Japan.

To assure the robustness of the results and to verify the magnitude of the potential biases owing to nonresponse, a multiple imputation method was used to impute the missing values. According to a recommendation by Royston and White,^21^ imputed data sets require at least 20 models in case 80% of cases have complete data on all relevant variables. Based on this suggestion, 20 models were created and merged to estimate the pooled odds ratios (ORs) and the 95% confidence intervals (95% CIs) of the outcome variables. All variables were used as predictors in the models. The missing rates in all the variables ranged from 0.2% to 11.2%.

Unconditional logistic regression analyses were then performed to calculate the adjusted ORs (aORs) for the association between working hours and obstetric and perinatal outcomes according to the monthly frequency of night shifts (during the first and second/third trimesters). Adjustments were made for age (younger than 20 years, 20‐29 years, 30‐39 years, or 40 years or older), educational background of the mother and her partner (junior high school/high school, vocational high school/technical school/junior college, university, or graduate school), household income (million Japanese yen) (<2 [approx. 18 000 US dollars], 2 to <4, 4 to <6, 6 to <8, 8 to <10, or ≥10), smoking status (never smoker, ex‐smoker [stopped before becoming aware of being pregnant], ex‐smoker [stopped after becoming aware of being pregnant], or current smoker), alcohol use (never drinker, ex‐drinker, current drinker [very little], current drinker [<150 g/wk as ethanol], current drinker [<300 g/wk], current drinker [<450 g/wk], or current drinker [≥450 g/wk]), psychological distress (score on Kessler‐6 scale: <9 or ≥9),^22^ body mass index (<18.5, 18.5 to <25, or ≥25 kg/m^2^), parity, in vitro fertilization and embryo transfer, and history of pregnancy loss. With respect to small‐for‐gestational‐age babies, we added the additional covariate of gestational weeks. Pregnancies resulting in miscarriage and induced abortions were excluded from the analyses except for threatened miscarriages and threatened preterm labor. Women who had experienced cesarean birth deliveries were removed from analyses assessing the risk of cesarean birth. To prevent multiple comparisons possibly yielding false‐positive findings, we adopted the Benjamini‐Hochberg method and assessed statistical significance by obtaining *q*‐values of the false detection rates. All statistical analyses were performed using IBM SPSS Statistics for Windows, version 24.0 (IBM Corp.).

The JECS protocol was approved by the Ministry of the Environment's Institutional Review Board on Epidemiological Studies (no. 100910001) and by the Ethics Committees of all participating institutions. Written informed consent was obtained from all the study participants.

## 3 RESULTS

After excluding women with multiple pregnancies and those without information on singleton or multiple pregnancies, 99 744 singleton pregnancies were included in the present analysis. The age (mean ± standard deviation) at registration was 30.7 ± 5.1 years (range: 14‐48). Of the women who specified their work, 52.8% (n* = *50 560) worked more than one hour per week during their second/third trimesters (Table 1). The three leading occupations on awareness of their pregnancy were office clerks (16.9%), nurses (10.6%), and attendants/waitresses (8.6%). Compared with nonworking women, working women were more likely to be in their 30s, primigravidas, and healthier in terms of body mass index before pregnancy, smoking status, and psychological mood. They were also more likely to have higher socioeconomic backgrounds (higher education levels and higher annual household income) (Table 1). These factors and ones revealing significant differences between working women and nonworking women were adjusted in the following multivariate logistic regression analyses.

**Table 1:** Demographic characteristics of pregnant women in their second/third trimesters, Japan, 2011‐2014^a^, ^b^, ^c^

Variables	Total	Nonworkers	Workers	*P*
	n = 95 721	n = 45 161	n = 50 560	
	n (%) or Mean ± SD	n (%) or Mean ± SD	n (%) or Mean ± SD	
Age (y)				
<20	985 (1.1)	732 (1.7)	253 (0.5)	<.001
20‐29	36 414 (39.5)	17 788 (40.8)	18 626 (38.4)	
30‐39	51 420 (55.8)	23 450 (53.9)	27 970 (57.6)	
≥40	3291 (3.6)	1576 (3.6)	1715 (3.5)	
Prepregnancy body mass index (kg/m^2^)				
<18.5	15 455 (16.2)	7581 (16.8)	7874 (15.6)	<.001
18.5‐<25.0	69 882 (73.1)	32 378 (71.8)	37 504 (74.3)	
≥25.0	10 207 (10.7)	5106 (11.3)	5101 (10.1)	
Education level				
Junior high school/high school	34 623 (36.3)	18 981 (42.3)	15 642 (31.0)	<.001
Vocational high school/technical school/ junior college	40 098 (42.1)	17 585 (39.2)	22 513 (44.7)	
University	19 202 (20.1)	7949 (17.7)	11 253 (22.3)	
Graduate school	1392 (1.5)	400 (0.9)	992 (2.0)	
Partner's education level				
Junior high school/high school	41 861 (44.2)	20 089 (45.0)	21 772 (43.4)	<.001
Vocational high school/technical school/ junior college	21 269 (22.5)	9327 (20.9)	11 942 (23.8)	
University	27 327 (28.8)	13 061 (29.3)	14 266 (28.5)	
Graduate school	4272 (4.5)	2133 (4.8)	2139 (4.3)	
Annual household income (million Japanese yen)				
<2	5041 (5.7)	3031 (7.3)	2010 (4.2)	<.001
2‐<4	30 857 (34.6)	17 761 (42.8)	13 096 (27.5)	
4‐<6	29 454 (33.1)	13 971 (33.6)	15 483 (32.5)	
6‐<8	14 175 (15.9)	4290 (10.3)	9885 (20.8)	
8‐<10	5802 (6.5)	1279 (3.1)	4523 (9.5)	
≥10	3786 (4.2)	1189 (2.9)	2597 (5.5)	
Smoking status				
Never smoker	54 859 (57.8)	24 690 (55.2)	30 169 (60.1)	<.001
Ex‐smoker, stopped before learning of pregnancy	22 736 (23.9)	11 464 (25.6)	11 272 (22.5)	
Ex‐smoker, stopped on awareness of pregnancy	12 976 (13.7)	6353 (14.2)	6623 (13.2)	
Current smoker	4373 (4.6)	2242 (5.0)	2131 (4.2)	
Alcohol use				
Never drinker	31 822 (33.5)	15 586 (34.8)	16 236 (32.3)	<.001
Ex‐drinker	60 572 (63.7)	28 067 (62.6)	32 505 (64.7)	
Current drinker	2 691 (2.8)	1197 (2.7)	1494 (3.0)	
Psychological distress^d^				
<9	85 797 (89.8)	39 767 (88.3)	46 030 (91.2)	<.001
≥9	9744 (10.2)	5285 (11.7)	4459 (8.8)	
Pregnancy loss history				
0	73 187 (77.2)	33 797 (75.6)	39 390 (78.7)	<.001
1	16 848 (17.8)	8434 (18.9)	8414 (16.8)	
2	3695 (3.9)	1902 (4.3)	1793 (3.6)	
≥3	1063 (1.1)	580 (1.3)	483 (1.0)	
In vitro fertilization and embryo transfer				
No	92 732 (96.9)	43 764 (96.9)	48 968 (96.9)	.574
Yes	2953 (3.1)	1378 (3.1)	1575 (3.1)	
Parity				
0	37 609 (40.2)	14 956 (33.8)	22 653 (46.0)	<.001
≥1	55 867 (59.8)	29 296 (66.2)	26 571 (54.0)	
Gestational age (wk)	38.8 ± 1.6	38.8 ± 1.6	38.9 ± 1.6	<.001

a Chi‐square or Student *t* tests were used to test for differences between nonworkers and workers.

b Working hours and frequencies of night shifts were measured in the second/third trimesters.

c Missing values are included in totals but not shown separately.

d The cutoff point was 9, with scores of 9 or higher indicating psychological distress.

Work during the first trimester generally increased the risk of threatened miscarriage and threatened preterm labor, with the aORs generally increasing in a time‐dependent manner (Table 2). However, a significantly increased aOR was not detected for actual pregnancy loss, for which the aOR could not be calculated owing to the low frequencies in some working‐hour categories under the present sample size. Significantly increased aORs were also not observed for actual preterm labor except for those working 1‐35 hours per week with night shifts 1‐6 times per month, for which the aORs for preterm labor (<37 and <34 weeks) were 1.40 (95% CI: 1.11‐1.76) and 1.76 (1.19‐2.61), respectively.

**Table 2:** Adjusted odds ratios (aORs) for the association between weekly working hours during the first trimester and pregnancy outcomes according to the monthly frequency of night‐shift work, Japan, 2011‐2014^a^, ^b^, ^c^

Night‐shift work (d/mo)	0			1‐6			≥7		
Working hours (h/wk)	1‐35	36‐45	≥46	1‐35	36‐45	≥46	1‐35	36‐45	≥46
n	21 301‐21 382	21 712‐21 747	9673‐9694	2041‐2162	2293‐2317	1204‐1244	565‐581	1752‐1764	1328‐1341
	aOR (95% CI)	aOR (95% CI)	aOR (95% CI)	aOR (95% CI)	aOR (95% CI)	aOR (95% CI)	aOR (95% CI)	aOR (95% CI)	aOR (95% CI)
Obstetric outcome of mothers									
Threatened miscarriage n = 11 950	**1.10 (1.04**‐**1.16)**	**1.24 (1.17**‐**1.31)**	**1.24 (1.15**‐**1.33)**	0.99 (0.83‐1.19)	**1.33 (1.16**‐**1.51)**	**1.41 (1.19**‐**1.67)**	1.16 (0.90‐1.50)	**1.32 (1.15**‐**1.53)**	**1.47 (1.26**‐**1.73)**
Threatened preterm labor n = 19 252	**1.07 (1.02**‐**1.12)**	**1.28 (1.22‐1.34)**	**1.32 (1.25‐1.40)**	0.94 (0.81**‐**1.09)	**1.51 (1.36‐1.68)**	**1.63 (1.41‐1.87)**	1.20 (0.97**‐**1.48)	**1.62 (1.45‐1.82)**	**1.63 (1.42‐1.86)**
Preterm labor (<37 weeks)^d^ n = 4832	1.03 (0.95**‐**1.11)	1.00 (0.92**‐**1.09)	0.99 (0.88**‐**1.10)	**1.40 (1.11‐1.76)**	0.90 (0.72**‐**1.12)	1.03 (0.77**‐**1.37)	1.24 (0.86**‐**1.77)	1.10 (0.87**‐**1.38)	1.07 (0.82**‐**1.39)
Preterm labor (<34 weeks)^d^ n = 1152	1.01 (0.86**‐**1.19)	0.98 (0.82**‐**1.16)	1.00 (0.80**‐**1.26)	**1.76 (1.19‐2.61)**	0.83 (0.53**‐**1.31)	0.97 (0.53**‐**1.78)	1.51 (0.80**‐**2.83)	1.11 (0.70**‐**1.75)	0.63 (0.32**‐**1.24)
Mild hypertensive disorders of pregnancy^d^ n = 2241	1.07 (0.95**‐**1.20)	1.06 (0.94**‐**1.20)	1.20 (1.03**‐**1.40)	1.20 (0.84**‐**1.70)	1.28 (0.98**‐**1.68)	1.40 (0.97**‐**2.01)	1.21 (0.73**‐**2.00)	1.27 (0.93**‐**1.72)	1.10 (0.76**‐**1.58)
Gestational diabetes mellitus^d^ n = 2652	0.88 (0.79**‐**0.98)	0.92 (0.82**‐**1.02)	0.82 (0.71**‐**0.96)	1.04 (0.75**‐**1.45)	0.91 (0.70**‐**1.20)	0.61 (0.39**‐**0.96)	0.81 (0.46**‐**1.42)	0.91 (0.67**‐**1.25)	0.94 (0.66**‐**1.34)
Meconium–stained amniotic fluid^d^ n = 3204	0.96 (0.87**‐**1.06)	**0.82 (0.75‐0.91)**	0.85 (0.75**‐**0.97)	0.81 (0.57**‐**1.14)	0.89 (0.70**‐**1.13)	0.87 (0.63**‐**1.20)	0.59 (0.33**‐**1.06)	**0.67 (0.50‐0.90)**	0.66 (0.47**‐**0.92)
Modes of delivery (vs normal)									
Vacuum/forceps^d^ n = 5666	1.05 (0.97**‐**1.15)	**1.27 (1.17‐1.38)**	**1.34 (1.22‐1.48)**	1.02 (0.80**‐**1.31)	**1.30 (1.09‐1.56)**	1.02 (0.79**‐**1.33)	1.11 (0.77**‐**1.59)	1.15 (0.94**‐**1.41)	1.17 (0.94**‐**1.46)
Cesarean^d^, ^e^ n = 10 336	1.03 (0.97**‐**1.09)	0.99 (0.94**‐**1.06)	1.10 (1.02**‐**1.19)	1.07 (0.89**‐**1.30)	1.11 (0.96**‐**1.28)	0.98 (0.80**‐**1.19)	1.16 (0.89**‐**1.51)	1.12 (0.97**‐**1.31)	0.93 (0.78**‐**1.12)
Perinatal outcome of children									
Small‐for‐gestational‐age baby^d^ n = 9723	1.06 (1.00**‐**1.13)	1.04 (0.98**‐**1.10)	0.99 (0.92**‐**1.07)	1.10 (0.93**‐**1.30)	1.12 (0.98**‐**1.30)	**1.32 (1.10‐1.59)**	0.98 (0.74**‐**1.31)	1.14 (0.97**‐**1.33)	0.98 (0.81**‐**1.18)
Low birthweight^f^ n = 8214	1.09 (1.01**‐**1.17)	1.06 (0.98**‐**1.14)	1.04 (0.94**‐**1.15)	1.19 (0.94**‐**1.52)	1.15 (0.95**‐**1.39)	1.26 (0.98**‐**1.63)	1.27 (0.91**‐**1.77)	1.25 (1.02**‐**1.53)	1.18 (0.94**‐**1.49)

Note

Reference: Nonworkers (n = 37 353**‐**37 406).

a Analyses were adjusted for age, educational background (mother and partner), household income, smoking, alcohol use, psychological distress, body mass index, parity, in vitro fertilization and embryo transfer, and history of pregnancy loss.

b Values in bold: *q* < 0.05 (equal to *P* < .0077), adjusted using the Benjamini‐Hochberg method for false detection rate.

c See text for results of analyses of other obstetric outcomes not listed in the table. The following five outcomes with the expected frequencies of <5 in each of the nine work‐pattern categories were excluded from analyses: pregnancy loss (≥12), severe hypertensive disorder of pregnancy, placenta previa, polyhydramnios, and placental abruption.

d Pregnancies resulting in miscarriage and induced abortion were excluded from analysis.

e Women who had experienced cesarean birth deliveries were excluded from analysis.

f Gestational week was added as an additional covariate.

Increased working hours during the first trimester increased the aOR for vacuum/forceps delivery (Table 2). The aOR increased in a time‐dependent manner in women without night shifts and was statistically significant for those working ≥36 hours per week. However, significantly increased aORs were not observed in those working ≥46 hours per week with night shifts 1‐6 times per month or in women working with night shifts ≥7 times per month. As for the perinatal outcomes of the children, aOR for small‐for‐gestational‐age babies was significantly increased (1.32 (1.10‐1.59)) only in women working ≥46 hours a week with night shifts 1‐6 times per month.

In contrast to the increased ORs as mentioned above, the aORs for meconium‐stained amniotic fluid were significantly decreased in women working 36‐45 hours per week without or with night shifts ≥7 times per month (0.82 (0.75‐0.91) and 0.67 (0.50‐0.90), respectively). As for other analyzed outcomes than ones listed in Table 2, premature rupture of membranes (n = 8688), nonreassuring fetal status (n = 2411), oligohydramnios (n = 1264), and prolonged labor (n = 3864) did not show significantly increased or decreased aORs in any work‐pattern categories (data not shown).

Working hours and the frequency of night shifts during the second/third trimesters had similar impacts on obstetric outcomes (Table 3). The aORs for threatened preterm labor increased in a time‐dependent manner up to around 1.6 (maximum aOR 1.61 (1.40‐1.85)), which appeared to be the peak, and were statistically significant in pregnant women working ≥36 hours per week with or without night shifts. However, the aOR for the actual preterm labor (<37 weeks) was significantly higher only in those working 1‐35 hours per week with night shifts 1‐5 times per month (2.99 (2.48‐3.60)) (Table 3). The aOR for vacuum/forceps increased in a time‐dependent manner in women without night shifts and became significant in those working ≥36 hours per week. However, significantly increased aORs were not observed in those working night shifts (Table 3). The aORs for meconium‐stained amniotic fluid were significantly lower in women working ≥46 hours per week without night shifts (0.82 (0.72‐0.94)).

**Table 3:** Adjusted odds ratios (aORs) for the association between weekly working hours during the second/third trimesters and pregnancy outcomes according to the monthly frequency of night‐shift work, Japan, 2011**‐**2014^a^, ^b^, ^c^

Night‐shift work (d/mo)	0			1**‐**5			≥6		
Working hours (h/wk)	1**‐**35	36**‐**45	≥46	1**‐**35	36**‐**45	≥46	1**‐**35	36**‐**45	≥46
n	18 992**‐**19 131	19 908**‐**19 932	7970**‐**7998	2025**‐**2141	1425**‐**1451	783**‐**803	405**‐**419	1154**‐**1161	796**‐**804
	aOR (95% CI)	aOR (95% CI)	aOR (95% CI)	aOR (95% CI)	aOR (95% CI)	aOR (95% CI)	aOR (95% CI)	aOR (95% CI)	aOR (95% CI)
Obstetric outcome of mothers									
Threatened preterm labor n = 19 252	1.02 (0.98**‐**1.07)	**1.25 (1.20‐1.31)**	**1.32 (1.24‐1.40)**	0.86 (0.75**‐**1.00)	**1.46 (1.29‐1.67)**	**1.51 (1.27‐1.79)**	1.03 (0.80**‐**1.33)	**1.61 (1.40‐1.85)**	**1.57 (1.33‐1.85)**
Preterm labor (<37 wk)^d^ n = 4832	1.04 (0.96**‐**1.13)	0.92 (0.85**‐**1.00)	1.01 (0.90**‐**1.14)	**2.99 (2.48‐3.60)**	0.95 (0.72**‐**1.25)	0.83 (0.57**‐**1.22)	1.01 (0.62**‐**1.65)	0.95 (0.71**‐**1.27)	1.16 (0.84**‐**1.61)
Mild hypertensive disorders of pregnancy^d^ n = 2241	1.03 (0.92**‐**1.17)	1.15 (1.02**‐**1.29)	1.17 (0.99**‐**1.37)	0.87 (0.56**‐**1.34)	**1.56 (1.14‐2.12)**	**2.02 (1.39‐2.93)**	1.16 (0.63**‐**2.13)	1.14 (0.78**‐**1.67)	1.07 (0.67**‐**1.71)
Gestational diabetes mellitus^d^ n = 2652	0.91 (0.81**‐**1.01)	0.88 (0.79**‐**0.98)	**0.75 (0.63‐0.88)**	0.85 (0.55**‐**1.32)	0.74 (0.51**‐**1.06)	0.66 (0.39**‐**1.13)	0.92 (0.50**‐**1.70)	0.87 (0.59**‐**1.26)	0.81 (0.50**‐**1.31)
Meconium‐stained amniotic fluid^d^ n = 3204	0.91 (0.82**‐**1.00)	0.88 (0.79**‐**0.97)	**0.82 (0.72‐0.94)**	0.69 (0.45**‐**1.04)	0.71 (0.51**‐**0.98)	0.83 (0.55**‐**1.24)	0.96 (0.55**‐**1.65)	0.95 (0.69**‐**1.30)	0.66 (0.43**‐**1.02)
Modes of delivery (vs normal)									
Vacuum/forceps^d^ n = 5666	1.09 (1.01**‐**1.19)	**1.24 (1.15‐1.34)**	**1.33 (1.21‐1.47)**	1.13 (0.87**‐**1.47)	1.19 (0.94**‐**1.49)	1.02 (0.74**‐**1.42)	1.54 (1.06**‐**2.23)	1.20 (0.94**‐**1.53)	1.39 (1.05**‐**1.83)
Cesarean^d^, ^e^ n = 10 336	1.03 (0.97**‐**1.10)	0.99 (0.93**‐**1.05)	1.04 (0.97**‐**1.13)	**1.42 (1.17‐1.72)**	1.10 (0.92**‐**1.31)	0.84 (0.65**‐**1.08)	1.13 (0.82**‐**1.54)	1.05 (0.87**‐**1.27)	1.22 (0.99**‐**1.51)
Perinatal outcome of children									
Small‐for‐gestational‐age baby^d^ n = 9723	1.06 (1.00**‐**1.12)	1.04 (0.98**‐**1.11)	1.01 (0.93**‐**1.10)	1.18 (0.97**‐**1.43)	1.21 (1.02**‐**1.44)	1.28 (1.02**‐**1.60)	1.06 (0.76**‐**1.47)	1.09 (0.90**‐**1.32)	1.11 (0.89**‐**1.39)
Low birthweight^f^ n = 8214	1.11 (1.03**‐**1.19)	1.03 (0.96**‐**1.12)	1.06 (0.95**‐**1.18)	1.24 (0.95**‐**1.63)	1.06 (0.84**‐**1.33)	1.15 (0.85**‐**1.56)	1.62 (1.12**‐**2.35)	1.24 (0.97**‐**1.58)	1.28 (0.97**‐**1.69)

Note

Reference: Nonworkers (n = 45 472**‐**45 531).

a Analyses were adjusted for age, educational background (mother and partner), household income, smoking, alcohol use, psychological distress, body mass index, parity, in vitro fertilization and embryo transfer, and history of pregnancy loss.

b Values in bold: *q* < 0.05 (equal to *P* < .0077), adjusted using the Benjamini‐Hochberg method for false detection rate.

c See text for results of analyses of other obstetric outcomes not listed in the table. The following seven outcomes with the expected frequencies of less than 5 in each of the nine work‐pattern categories were excluded from analyses: preterm labor (<34 wk), pregnancy loss (≥12), severe hypertensive disorder of pregnancy, placenta previa, oligohydramnios, polyhydramnios, and placental abruption.

d Pregnancies resulting in miscarriage and induced abortion were excluded from analysis.

e Women who had experienced cesarean births were excluded from analysis.

f Gestational week was added as an additional covariate.

The following outcomes showed different associations from those with working hours according to the frequency of night shifts during the first trimester. Mild hypertensive disorders of pregnancy showed significantly increased aORs in association with increasing working hours during second/third trimesters. In women working night shifts 1‐5 times per month, the aOR increased in a time‐dependent manner to 2.02 (1.39‐2.93) and became significant for ≥36 hours per week. Cesarean birth revealed a significantly increased aOR (1.42 (1.17‐1.72)) only in those working 1‐35 hours per week with night shifts 1‐5 times per month (Table 3). In contrast to the increased risks mentioned above, time‐dependent decreases in aOR were observed for gestational diabetes, with a significant difference in women working ≥46 hours per week without night shifts (aOR 0.75 (0.63‐0.88)). The aOR for small‐for‐gestational‐age babies, which was significantly increased with a work pattern in the first trimester, was not increased for women who worked in the second/third trimester (Table 3).

As for other analyzed outcomes than ones listed in Table 3, premature rupture of membranes, nonreassuring fetal status, and prolonged labor did not show significantly increased or decreased aORs in any work‐pattern categories (data not shown).

## 4 DISCUSSION

The present study found increased risks of specific adverse obstetric and perinatal outcomes for women with long working hours and more night shift work. Some work patterns during the first trimester were significantly associated with threatened miscarriage, threatened preterm labor, preterm labor, vacuum/forceps delivery, and small‐for‐gestational‐age babies. Similarly, some work patterns during the second/third trimesters were significantly associated with mild hypertensive disorders of pregnancy, threatened preterm labor, preterm labor, and vacuum/forceps and cesarean birth.

First, the risk of threatened miscarriage and threatened preterm labor was slightly higher in the working population in our target cohort. In this regard, working long hours (>35 hours per week), standing >2 hours, and physically strenuous working conditions are associated with increased threatened preterm birth rates.^23^, ^24^ The results of our present study were consistent with these findings. Such adverse effects might be alleviated depending on the social system of the country; for example, the effects of work‐related psychosocial strain on preterm delivery and low birthweight have not been observed in Denmark, which has a highly developed social support system.^25^ An elevated risk of actual pregnancy loss or preterm labor was not detected except in women working 1‐35 hours per week with night shifts 1‐6 (1‐5 for the second/third trimesters) times per month. A recent report suggested that night work was not associated with an elevated risk of preterm birth.^11^ In contrast, Begtrup et al^12^ suggested that night workers might have an increased risk of miscarriage compared with the risk in pregnant women not working night shifts. The result in the present study was concordant with that of the former report despite the increased risks of threatened miscarriage and threatened preterm labor, which might be a result of obstetricians prescribing complete rest for most pregnant women facing threatened miscarriage or threatened preterm labor with signs of genital bleeding. Among women working 1‐35 hours per week with night shifts 1‐6 times per month, the average number of working hours per day (6.78 ± 1.90, mean ± standard deviation during the first trimester) was longer than those for the same working hour categories with different night‐shift frequencies (5.61 ± 1.51 for no night shift and 6.37 ± 1.71 for those working night shifts ≥7 times per month), which might partially explain the increased risk of preterm labor in this category. After multiple imputation analysis, this category had a lower percentage of women in professional occupations (13%) such as nurses, doctors, and social workers, and a higher percentage of students (4%). In this category, the risk of cesarean birth was increased in association with work during the second/third trimester. Health management of women working part‐time jobs with night shifts during pregnancy should be reviewed because shorter weekly working hours often indicate nonregular employment.

Second, working ≥36 hours per week with night shifts during the second/third trimesters significantly increased the risk of the onset of mild hypertensive disorders of pregnancy. The aORs increased in a time‐dependent manner and working ≥46 hours with night shifts 1‐5 days per month doubled the risk. The increased risk, however, was not observed for those working night shifts ≥6 times per month. In these categories, the most representative occupation was nursing. The results suggest that better health management during pregnancy was available for nurses.

Third, the risk of vacuum/forceps delivery increased in a time‐dependent manner in women with or without night shifts in association with work during the first trimester, and in women without night shifts in association with work during the second/third trimesters. The increase was significant in women working ≥36 hours per week. An increase in the risk was also not observed for longer working hours with more frequent night shifts, in which the predominant occupation was nursing.

Fourth, the increased risk of small‐for‐gestational‐age babies was observed in women working ≥46 hours a week with night shifts 1‐6 times per month during the first trimester. Using a questionnaire completed at an average of 30.8 weeks of pregnancy, Snijder et al^26^ reported that compared with women working <25 hours per week, those working 25‐39 and >40 hours during mid‐pregnancy had lower fetal birthweights by 148‐198 g. Our data were consistent with these findings and suggested that working over 45 hours per week with night shifts during the first trimester might negatively affect fetal growth after the second trimester.

Nursing is a representative occupation requiring shift work in which women are involved. Nurses encounter biological, infectious, chemical, environmental, physical, and psychosocial exposures at work^14^, ^27^ and are at a higher risk for cesarean birth, tocolysis, and threatened preterm labor compared with nonnurses.^14^ In addition, Lawson et al^28^ suggested that occupational exposures common to nurses are related to their risks of spontaneous abortion. However, in the present study, increased risks of adverse pregnancy outcomes were not detected in categories in which nursing was the predominant occupation except for an increased risk of small‐for‐gestational‐age babies, as mentioned above.

Working women without night shifts had a significantly lower risk for gestational diabetes and meconium‐stained amniotic fluid than those in nonworking women. The work itself might have reduced the risk of these complications. Catalano et al^29^ reported that both maternal gestational diabetes and obesity were independently associated with adverse pregnancy outcomes and that their combination had a greater effect than either factor alone. Further studies are needed on factors such as diet and physical activity of working women to clarify which aspect of work plays a primary role in reducing the risks of gestational diabetes and meconium‐stained amniotic fluid.

The JECS, with 100 000 participants, represents the largest nationwide birth cohort study in Japan. The outcome measurements are reliable because pregnancy and delivery information are based on medical records transcribed by doctors, research coordinators, nurses, and midwives. However, this study had several limitations. Although sociodemographic and physiological factors were adjusted in the analyses, there may remain unmeasured confounders, such as those related to the healthy worker effect, or occupation‐related confounders. The reference group in the analyses was nonworking women. This group included former workers; 17% of whom at the time of the first questionnaire had been working on awareness of their pregnancy. Between the first and the second/third trimesters, about 14% of working women left their jobs. In addition, the majority of pregnant women were recruited after the latter half of the first trimester,^15^ which means that early miscarriages were not covered in the JECS. Thus, we did not analyze the risk of early miscarriages despite its importance as an occupational health issue.^9^, ^10^, ^12^

### 4.1 Conclusions

Pregnant workers had increased risks of threatened miscarriage and threatened preterm labor, although the risks of abortion and preterm labor did not increase except for those women working 1‐35 hours per week with night shifts 1‐6 (5) times per month. Working more than 35 hours per week was associated with an increased risk of vacuum/forceps delivery, and such work during the second/third trimesters increased the risk of mild hypertensive disorders of pregnancy. Although nursing work was not likely to be high‐risk, working more than 45 hours per week with night shifts during the first trimester increased the risk of small‐for‐gestational‐age babies. Health management of women working part‐time jobs with night shifts during pregnancy needs to be reviewed in the context of increased preterm labors and cesarean births. Women wanting to become pregnant are advised to be aware of the risks related to their work patterns.
